# A metabolomic investigation of the effects of vitamin E supplementation in humans

**DOI:** 10.1186/1743-7075-9-110

**Published:** 2012-12-19

**Authors:** Max Wong, John K Lodge

**Affiliations:** 1 Department of Paediatrics, University of Cambridge, Addenbrooke’s Hospital, Cambridge, CB2 0QQ, UK; 2 School of Life Sciences, Northumbria University, Ellison Building, Newcastle-Upon-Tyne, Tyne & Wear, NE1 8ST, UK; 3 Faculty of Health and Medical Sciences, University of Surrey, Guildford, Surrey, GU2 7XH, UK

**Keywords:** Metabolite profiling, LC/MS, Vitamin E, Lysophospholipids

## Abstract

**Background:**

Vitamin E is a nutrient with both antioxidant and non-antioxidant activities and has been shown to modulate the function of a number of cell types *in vitro* and in human studies. However studies have also shown vitamin E to have detrimental interactions and therefore it is important to establish the extent to which this nutrient influences metabolism. Metabolomics can potentially identify nutrient-metabolism interactions and therefore the aim of this study was to use a non-targeted metabolomic approach to identify changes to the plasma metabolome following vitamin E supplementation in humans.

**Methods:**

A relatively homogenous healthy adult male population (n = 10) provided a fasting blood sample immediately before and after a 4-week vitamin E supplementation regime (400 mg/d of *RRR*-α-tocopheryl acetate)) on top of their habitual diet. Plasma samples were analysed for vitamin E and clinical markers. Plasma underwent non-targeted metabolite profiling using liquid chromatography/mass spectroscopy and data was processed using multivariate statistical analysis.

**Results:**

Plasma vitamin E concentrations were significantly increased following supplementation (p < 0.001). A partial least squares-discriminant analysis (PLS-DA) model was able to discriminate between samples taken pre and post vitamin E supplementation (goodness of fit R^2^Y = 0.82, predictive ability Q^2^ = 0.50). Variable influence on projection and PLS-DA loadings highlighted a number of discriminating ions that were confirmed as discriminatory through pairwise analysis. From database searches and comparison with standards these metabolites included a number of lysophosphatidylcholine species (16:0, 18:0, 18:1, 18:2, 20:3 and 22:6) that were increased in intensity post supplementation by varying degrees from 4% to 29% with the greatest changes found for lysoPC 22:6 and 20:3.

**Conclusions:**

Although a small scale study, these results potentially indicate that vitamin E supplementation influences phospholipid metabolism and induces lysoPC generation; a general pro-inflammatory response. Moreover the study identifies novel areas of vitamin E interactions and highlights the potential of metabolomics for elucidating interactions between nutrients and metabolic pathways in nutritional research.

## Background

It is well established that nutrients interact with metabolic pathways at various levels and have multiple targets. With the increasing use of ‘-omic’ technologies in nutritional research [1] it is now becoming possible to further explore the influence of single nutrients or dietary change on physiological processes.

Vitamin E has been widely researched owing to its antioxidant [2] and non-antioxidant functions [3] and its potential as a cardioprotective agent. Vitamin E appears to have wide ranging effects on cellular systems with the ability to inhibit protein kinase C, activating diacylglycerol kinase (decreasing diacylglycerol availability) and protein phosphatase 2A [4], regulating specific gene expression [5,6]; in this way vitamin E can influence a number of biological functions and metabolism. Interestingly, α-TTP knock out mice (model of vitamin E deficiency) had lower glucose levels, improved glucose tolerance and increased insulin excretion [7] indicating a role for α-tocopherol regulation in glucose control. Vitamin E supplementation has been shown to influence platelet [8] and mononuclear cell functions [8,9], and reduce indices of oxidative stress and inflammation [10] in humans. However vitamin E may also have potential detrimental actions as high dose (>400 mg/d) supplementation was found to increase mortality in a meta-analysis [11] and high dose supplementation has been proposed to potentially interfere with drug metabolism [12]. As nutrients generally have multiple targets and interactions, it is likely that vitamin E has alternate functions and can further influence metabolic pathways.

Metabolomics offers the opportunity to investigate global changes to metabolites in biological samples and there is much interest in this technology for nutritional studies [1,13]. Potential opportunities include a further understanding of the interactions of whole diet or individual nutrients on metabolic pathways, the metabolic interactions between diet and disease, and developing biomarkers for dietary exposure and/or disease [14,15]. Metabolomic technology has been applied to a number of nutritional studies (reviewed in [1,16]), allowing investigators to compare the influence of dietary patterns on metabolite profiles [17], monitor nutrient metabolism or identify novel metabolic pathways influenced by the nutrient [18]. Interestingly metabolomics has previously been used in animal models to investigate vitamin E metabolism. Following pregnane X receptor activation in knockout mice there was an attenuation of α-tocopherol metabolism but production of a novel γ-tocopherol metabolite [19]. Novel vitamin E metabolites were also found by Johnson et al. in human and mouse models by LC/MS [20]. In a mouse model of neuronal ceroid lipofuscinosis, vitamin E supplementation reversed metabolic abnormalities associated with the phenotype [21]. In a rat liver model of vitamin E deficiency, the authors found metabolic changes associated with amino acids, glucose and purines that suggested shifts in energy metabolism with deficiency [22]. In the only human metabolic study involving vitamin E, metabolomics was successfully used to determine ‘predictors of response’ to vitamin E treatment in subjects with Non-Alcoholic Steatohepatitis [23]. These studies demonstrate that vitamin E can influence metabolic pathways and so it is of interest to investigate other potential interactions in a human supplementation study.

We have previously highlighted that metabolomics can be used to provide insights into how vitamin E can influence the metabolome [16] and in the present study we have expanded on these observations to identify subtle but distinct changes to human plasma metabolome following vitamin E supplementation, the main effects of which include significant increases in the intensities of a number of lysophospholipid species.

## Methods

### Materials

All solvents were purchased from Fisher Scientific (Loughborough, UK). Acetonitrile and methanol were LC/MS grade, formic acid was laboratory reagent grade. Water was purified using an Elga PureLab Ultra system (Elga, High Wycombe, UK). Leucine encephalin and lysophosphatidylcholine pure standards (analytical grade) were purchased from Sigma Aldrich (Poole, UK). Gelatine capsules containing 400 mg of natural (*RRR*) alpha-tocopheryl acetate (purity 98.9%) in vitamin E-stripped corn oil were purchased from Eurocaps Ltd. (Gwent, UK).

### Human study

For this study 10 male subjects were recruited from within the University of Surrey and surrounding area through advertisement. Their average age and BMI were 32 ± 9 years and 24 ± 4 kg/m^2^ respectively. The subjects were outwardly healthy, normolipidaemic (total cholesterol 4.76 ± 1.3 mmol/L and triacylglycerols 0.95 ± 0.6 mmol/L) and were not taken any prescription medication or dietary supplements. The study was a one-way supplementation trial with subjects taking a capsule containing 400 mg of alpha-tocopheryl acetate every day for 4 weeks on top of their habitual diet (no dietary standardisation was performed). At the start and end of the trial, subjects visited the clinical investigation unit at the University of Surrey in a fasted state where they provided a blood sample (20 mL, EDTA). Under identical conditions for each collection, plasma was harvested by centrifugation, immediately aliquoted (0.5 mL), frozen and stored at -80°C prior to analysis. The University of Surrey Ethics Committee granted approval for this study.

### Sample preparation

For protein precipitation, 100 μl of freshly thawed human plasma was vortex mixed with 400 μl of chilled (-80°C) methanol for 1 min then left on ice for 10 min. The mixture was then centrifuged at 13,000 rpm for 10 minutes and the supernatant transferred to autosampler vials (Waters, Manchester, UK) for LC/MS analysis. For metabolite identification, pure standards (1 ng/μL injected) were dissolved in methanol and analysed under the same conditions as for the plasma samples. Plasma samples were also spiked with pure standards.

### Liquid Chromatography Mass Spectrometry (LC/MS)

Chromatography was performed on a Waters Acquity UPLC™ system (Waters, Manchester, UK) using an Acquity BEH C_18_ column (1.7 μm 2.1 × 100 mm) for sample separation following a 5 μL injection volume using partial loop mode. The column oven was maintained at 40°C and the autosampler at 4°C. The mobile phase consisted of: (A) 0.1% formic acid in water, and (B) acetonitrile with 0.1% formic acid. The gradient program began with 100% (A) for 0.5 min, then proceeded with a linear gradient to 100% (B) over 8.5 min, then returned to initial conditions (100% A) and maintained for 1 min. The total run time was 10 min with a flow rate of 0.6 ml/min.

Mass Spectrometry was performed on a Waters Micromass QToF Premier™ (Waters, Manchester, UK) operating in both positive and negative ion electrospray modes. The source temperature was set to 120°C, the desolvation temperature was 450°C, with nebulisation gas set to 800 L/hr. The collision energy was set at 4 eV. The capillary and cone voltages were set at 3.1 kV and 38 V respectively in positive ionisation mode, and 2.9 kV and 35 V respectively in negative ionisation mode. A LockSpray™ interface was used to ensure mass accuracy. For this leucine-enkephalin (556.2771 m/z in positive ionisation mode) was infused at a concentration of 200 ρg/μL at a flow rate of 0.01 ml/min. Data was collected in centroid mode over the range 100 to 1000 m/z with an acquisition rate of 0.2 s, interscan delay of 0.02 s, with dynamic range enhancement activated. MS/MS experiments were performed by ramping the collision energy from 10–50 V on selected masses of interest while all other parameters remained the same. Each sample was injected 6 times. The sample list was randomised and also contained a quality control sample and a metabolite test mix sample which were analysed following every 20 injections in order to monitor retention time and mass drift within the total analytical period.

### Other analysis

Plasma vitamin E concentrations were analysed by HPLC incorporating electrochemical detection as previously described [24]. Plasma total cholesterol, triacylglycerols, glucose and nonesterified fatty acids were measured using enzymatic test kits (Instrumentation Laboratory, UK) on an automated clinical analyser (ILab650, Instrumentation Laboratory, UK).

### Data analysis

The acquired LC/MS data was analysed and visualised using MassLynx and MarkerLynx (both Version 4.1, Waters), using a noise elimination level set at 6 and 15 masses per retention time. Background ions from associated blank injections were excluded from each sample. The mass and retention time windows were 0.05 Da and 0.1 min respectively and signal drift was corrected automatically by the software. The peak information was then transferred to a multivariate analysis package (SIMCA-P+,Ver 12, Umetrics AB, Sweden). Data were initially analysed by Principal Component Analysis (PCA) to identify any outliers. All variables were set to Pareto scaling. A 2-component model was generated for Partial Least Squares Discriminant Analysis (PLS-DA) and the predictive ability (Q^2^) of the model assessed by internal cross validation (seven-group venetian blind cross-validation). A predictive set was established by randomly removing 30% of the data to form a training set and testing the predictive ability of the model against the remaining ‘test’ data through averaging seven iterations of the data. The model was able to correctly classify 72% of samples (in both groups) in a predictive set. PLS-DA loadings and variable influence on projection (VIP) plots were used to identify the most important discriminatory species from the total list of features detected. For the pairwise analysis of signal intensities, Wilcoxon Matched Pairs tests were performed using Prism (Ver 5, GraphPad Software Inc). A Bonferroni correction, to take into account multiple comparisons, was applied using the number of discriminating ions from the VIP plot as the number of variables in the calculation*,* giving an alpha level of 0.002. Only signals that were significantly different with treatment (p < 0.05), or a trend for significance (p < 0.1) following pair-wise comparisons, underwent putative identification.

## Results and discussion

In this study LC/MS based metabolomics has been applied to plasma samples obtained pre and post vitamin E supplementation. Metabolomics is being increasingly used in nutritional studies [16], as it is of importance to further understand how diet influences metabolic pathways and to develop biological markers of dietary exposure [14]. Vitamin E supplementation has been shown to have varied responses in a number of *ex vivo* and *in vitro* studies and with the development of more sophisticated research tools such as metabolomics, our aim was to use this technology to identify any effects of vitamin E supplementation on human metabolism. Vitamin E plasma concentrations were relatively high at baseline, suggesting the subjects were replete in vitamin E, but values significantly increased from 31.53 ± 4.6 μmol/L before supplementation to 46.65 ± 5.16 μmol/L post supplementation (p < 0.001), which represents a 1.5 fold increase in concentration. This is in line with what is expected from similar vitamin E interventions [25] as it is known that due to hepatic regulation plasma concentrations become saturated and cannot be raised more than 2–3 fold [26]. There were no changes to plasma concentrations of glucose, total cholesterol and triacylglycerol following supplementation (data not shown).

### Classification of samples

**Figure 1:**
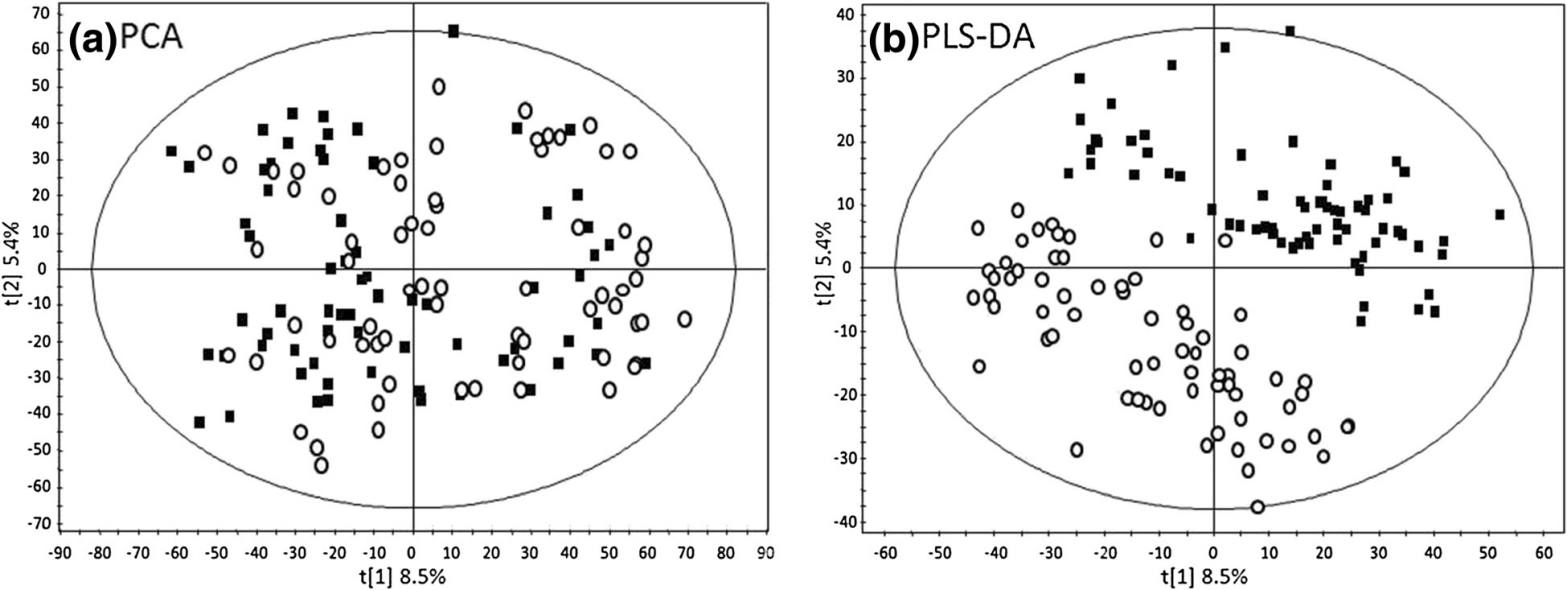
**Multivariate analysis of data.** (**a**) PCA and (**b**) PLS-DA scores plot showing samples pre (*black square*) and post (○) vitamin E supplementation.

The LC/MS approach detected approximately 2500 ion species in plasma samples, and there was no significant difference between the total number of species detected pre (2551 ± 176) and post (2556 ± 142) supplementation. The data were firstly analysed by unsupervised Principal Component Analysis (PCA) to check for overall data quality (Figure 1a). We found no data points outside the Hotelling confidence intervals and so no data points were investigated further. PCA was unable to discriminate between sample groups (Figure 1a), however this is not uncommon as PCA may not be sensitive enough to discriminate between samples sets when there is large variation and only modest differences between groups, as commonly seen in nutritional studies [27].

**Figure 2:**
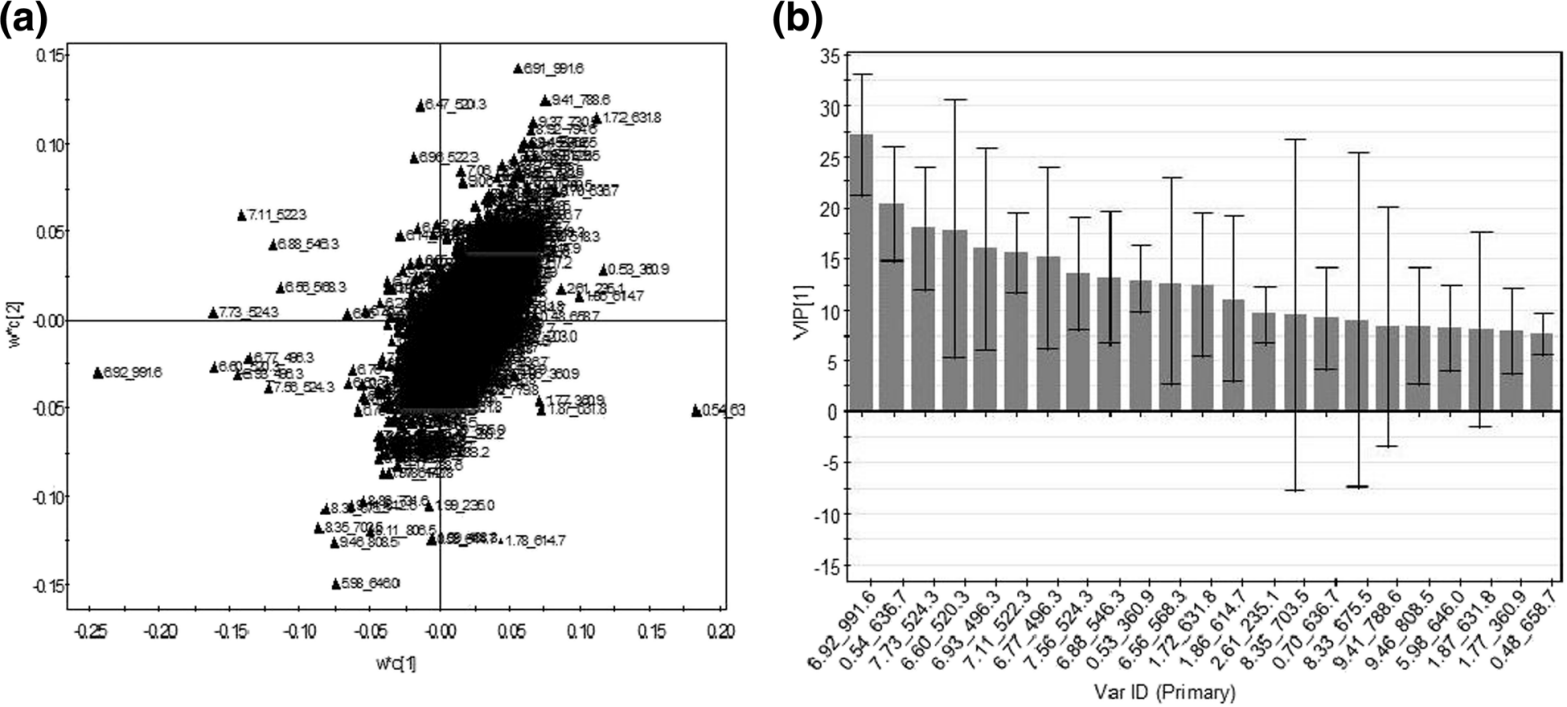
**Multivariate analysis of data.** (**a**) PLS-DA loadings plot of first two latent variables explaining separation in Figure 1b, and (**b**) associated VIP plot of the first component highlighting discriminatory species.

To further explore the data a two-component PLS-DA model was established (Figure 1b), and was able to discriminate between treatment (model parameters; R^2^Y = 0.82, Q^2^ = 0.50), with samples post supplementation clustering mainly to the left along the first latent variable axis. To identify discriminatory species the data was explored through a PLS-DA loadings plot and a VIP plot of the first latent variable (Figure 2a and b respectively). This generated a list of top discriminatory species above a VIP score of 7.5. We then analysed single ion chromatograms and associated molecular ions of the species in the original data in order to check for adducts and remove false positive signals, and performed pairwise comparisons of ion intensities between sample class to confirm differences between treatment groups. From this analysis out of the approximately 2500 features initially detected, only seven discriminatory signals remained for further identification.

### Identification of signals discriminatory for vitamin E supplementation

**Figure 3:**
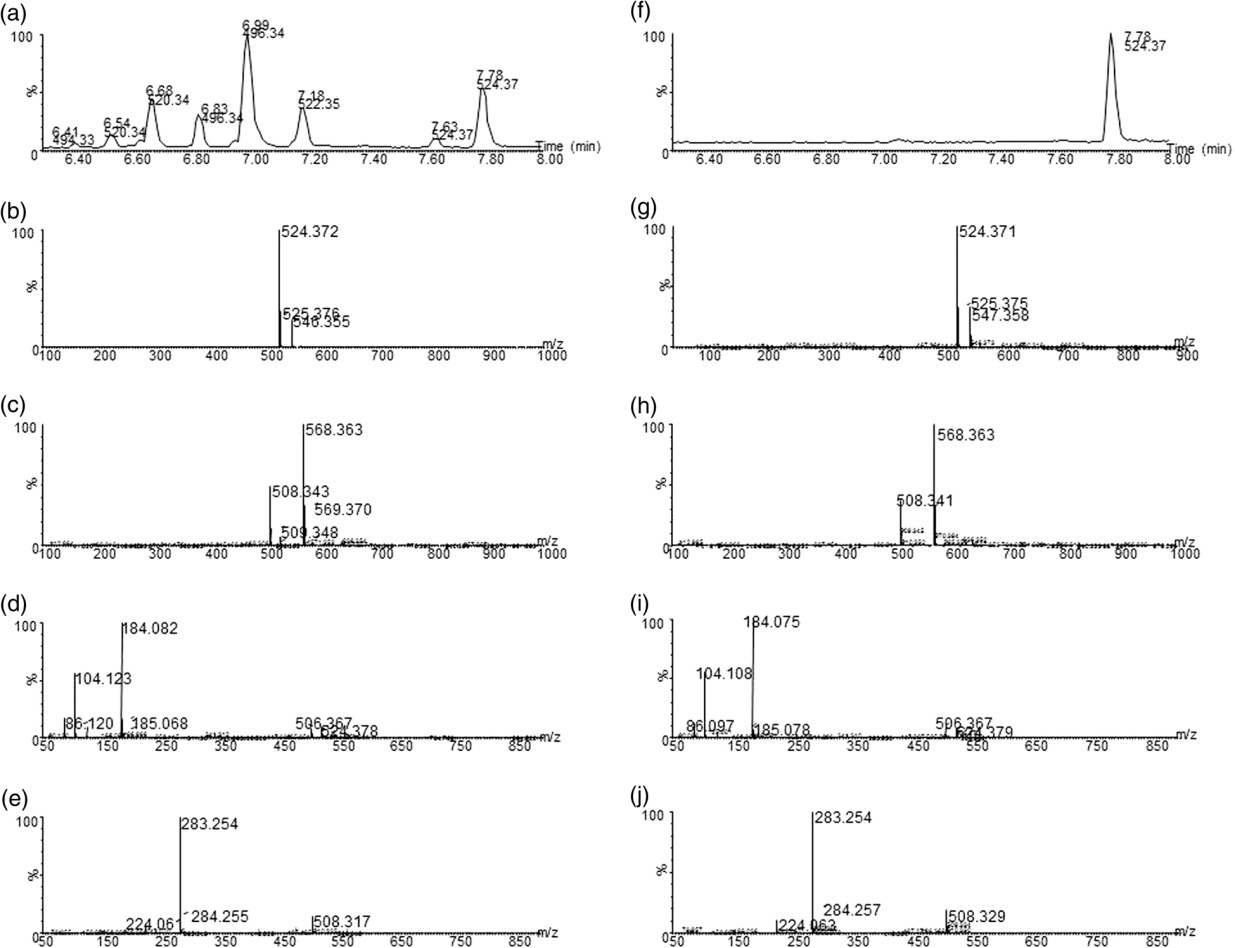
**Mass spectroscopy of the peak at 7.78 min in a representative plasma sample (left panels (a)-(e)) and a pure standard of lysophosphatidylcholine (C18:0) (right panels (f)-(j)).** Panels (**a**) & (**f**) show a TIC of the region 6.3-8 mins. A spectrum of the peak at 7.78 min is shown in panels (**b**) & (**g**) and panels (**c**) & (**h**) in positive and negative ion modes respectively. MS/MS spectrums of the same peak are shown in panels (**d**) & (**i**) and (**e**) & (**j**) for positive and negative ion modes respectively.

**Figure 4:**
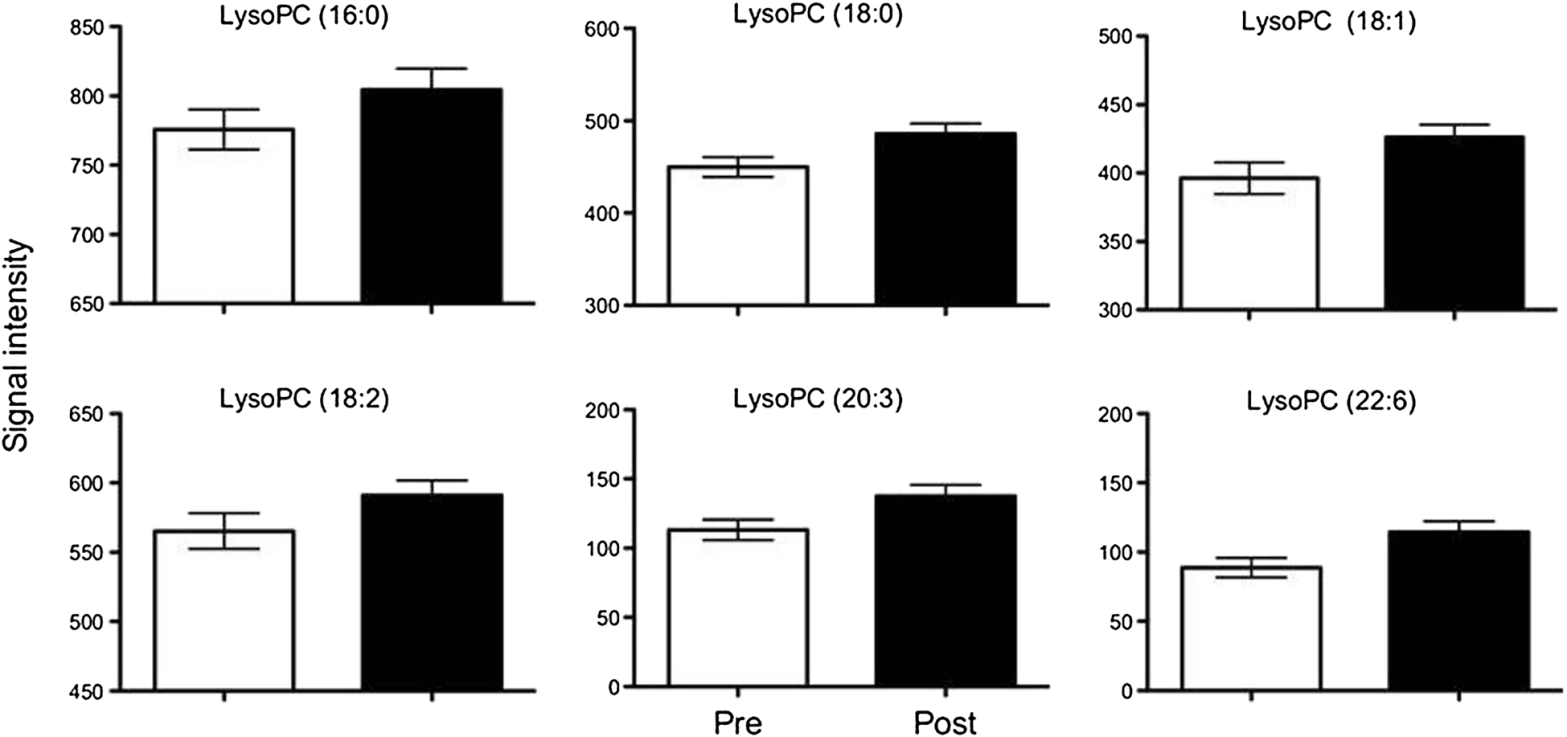
**Increase in intensity of various lysophosphatidylcholine species post vitamin E supplementation.** Data shown as mean ± SEM signal intensity in samples taken pre (open boxes) and post (closed boxes) supplementation.

To identify these discriminatory species, elemental composition analysis was performed using Masslynx to obtain potential formulae. Masses were then entered into metabolite databases (Human Metabolite Database, http://www.hmdb.ca; METLIN, metlin.scripps.edu), and formulae compared with possible elemental composition taking into account potential adducts from molecular ion analysis. To confirm identities pure compounds were purchased when possible and analysed under identical conditions to that of the plasma samples, including spiking of original samples. Molecular ion and fragmentation patterns of the standards and the associated peak in original samples, retention time and sample spiking confirmed the assignments. An example of such an assignment strategy is shown in Figure 3 for the species at 7.78 min, 524.37 m/z in plasma. A positive ion mode TIC of the region around the peak in a representative plasma sample is shown in Figure 3a. A spectrum of the peak at 7.78 min in positive mode (Figure 3b) revealed a major signal at 524.372 m/z and in negative mode (Figure 3c) at 568.36 and 508.34 m/z. The top match for this signal from metabolite databases was found to be the [M + H]^+^ adduct of lysophosphatidylcholine (C18:0). MS/MS fragmentation patterns of the peak at 7.78 min in positive mode (Figure 3d) revealed major signals at 184.07 and 104.10 m/z characteristic of the phosphocholine moiety [28] and in negative mode (Figure 3e) at 283.25 m/z characteristic of the [M-H]^-^ adduct of stearic acid. A comparison of this analysis with that of a pure standard of lysophosphatidylcholine (C18:0) (Figure 3f-j) revealed identical mass spectroscopy behaviour. Using the same approach, six lysophosphatidylcholine (LysoPC) species were assigned (Table 1) that were increased in intensity post supplementation (pairwise analysis, Figure. 4). Similar assignment strategies were used for the confirmation of LysoPC in previous studies [29,30].

**Table 1:** Major discriminatory species identified from the PLS-DA loadings and VIP plot

**VIP**	***m/z *[M + H]**^**+**^	**RT (min)**	*P *value*	**Assignment**
18.02	524.371	7.78	0.037	LysoPC (18:0)
17.93	520.340	6.60	0.052	LysoPC (18:2)
16.02	496.339	6.93	0.085	LysoPC (16:0)
15.67	522.355	7.11	0.045	LysoPC (18:1)
13.32	546.356	6.88	0.018	LysoPC (20:3)
12.73	568.340	6.56	0.023	LysoPC (22:6)
9.59	235.181	2.61	0.012	NA

* from pairwise comparisons pre vs post, with an adjusted Bonferroni p value (<0.002) no significance remained. NA, no assignment from metabolite databases. All signals were increased in intensity post vitamin E supplementation.

Other discriminatory species influenced by vitamin E supplementation remain to be assigned as no matches were found on metabolite databases. As vitamin E metabolites appear in the plasma and urine following supplementation [31] it may have been expected to find such metabolites as discriminatory species in the current study. The α-tocopherol metabolites α-carboxyethyl hydroxychroman (α-CEHC) and its glucuronide derivative were found to be discriminating in a metabolomic investigation in mice following activation of the pregnane X nuclear receptor [19]. However, molecular ions of these species and their potential adducts were not found in the current study, presumably a consequence of the protein precipitation technique used.

### Lysophophatidylcholine species are significantly increased in response to vitamin E supplementation

The intensity of lysoPC species were increased varyingly; 22:6 (29%), 20:3 (21%), 18:0 (8%), 18:1 (7.5%), 18:2 (4.5%), and 16:0 (4%). LysoPC are products of phospholipid metabolism formed predominantly through the action of phospholipase A_2_ (PLA_2_) activity on biological membranes, but also through Lecithin Cholesterol Acyl Transferase (LCAT) activity between lipoproteins, and during the oxidative modification of LDL [32]. When LysoPC are formed there is a loss of a fatty acid molecule and so one might expect levels of nonesterified fatty acids (NEFA) to increase in the plasma. We measured NEFA in pre and post samples and found NEFA to be increased non-significantly following vitamin E supplementation (0.189 ± 0.08 mmol/L in pre vs 0.202 ± 0.11 mmol/L in post). LysoPC have specific actions and are not simply a consequence of phospholipid metabolism. LysoPC have pro-inflammatory activity, having the ability to activate a variety of cell lines and this activation has been shown to be dependent on the acyl chain length, with saturated species being the most effective [33]. LysoPC are mediators of endothelial function [32], capable of inducing endothelial dysfunction, platelet activation [34], and migration of smooth muscle cells [35]. Some of these lysoPC actions appear to be mediated through signal-regulated kinases [34], the release of growth factors [35] and through binding to specific receptors [32]. A release of lysoPC following vitamin E supplementation would suggest a general pro-inflammatory response, however vitamin E is thought to have anti-inflammatory properties. Vitamin E has been shown to attenuate the pro-inflammatory action of lysoPC in various cell culture studies [34-36]. For example, a recent study in high cardiovascular risk subjects demonstrated an overall anti-inflammatory effect following long-term vitamin E supplementation [10]. Opposing roles for vitamin E can occur; the simultaneous potentiation of PLA_2_ activity but inhibition of COX activity favoured production of vasodilator prostanoids in endothelial cells [37], and this may provide an overall beneficial effect in certain tissues and provide an explanation for these contradictory findings.

It is interesting to note that impaired vitamin E regulation appears to play a role in glucose control through unknown mechanisms [7]. The finding that lysoPC are involved in both adipocyte glucose uptake [38] and glucose-dependent insulin secretion [39], and that lyso-PC have been shown to be potential biomarkers of type 2 diabetes [40] does link these various lines of evidence and warrants further study.

### Potential mechanisms of raised lysophosphatidylcholines

There are a number of potential mechanisms by which vitamin E supplementation could increase levels of lysoPC. Vitamin E has been shown to influence gene expression of a number of systems and modulate the activities of a number of enzymes involved in signal transduction, including PLA_2_[41]. Depending on the model used vitamin E treatment has been shown to activate [37,42,43], inhibit [44-46] or have no effect [47] on PLA_2_ activity, but the weight of evidence overall is suggestive of an increase in PLA_2_ activity following vitamin E exposure in cell/animal models. Because of the difference in models used and lack of human data it is not possible to relate PLA_2_ activity with vitamin E levels. Vitamin E can form complexes with lysoPC which help to maintain membrane structure and although vitamin E is thought to stabilise membranes through its interaction with phospholipids and other membrane components, there is evidence that it can also destabilise [48]. An increase in vitamin E content of biological membranes, following supplementation, may potentially disrupt the membrane causing displacement of membrane phospholipids, which are metabolised releasing lysoPC. However it is not known if a 1.5 fold increase in vitamin E content (as found in the current study) could have these effects on biological membranes. Alternatively the result may not necessarily be specific to vitamin E but could be representative of an acute phase response to the intervention, or a similar phenomenon, as other studies have found lysoPC to be discriminating species. For example, a metabolomic study found a number of lysoPC species increased during an oral glucose tolerance test [18]. LysoPC have also been associated with a number of disease states and found to be increased in subjects with hepatitis B-induced deterioration of liver function [29], rheumatoid arthritis [49], type 2 diabetes [50] and obesity [51], whereas lysoPC were significantly decreased in subjects with colorectal cancer [52], and in sepsis patients [53]. Therefore, as lysoPC appear to be influenced by a number of physiological conditions further work is necessary to elucidate the mechanism of this effect and the identification of other species influenced by vitamin E supplementation. It would be of interest to pursue and validate these observations in a more comprehensive study of vitamin E supplementation.

## Conclusions

Overall the data in this study suggest that vitamin E supplementation influences phospholipid metabolism, increasing the intensity of a variety of lysophosphatidylcholine species in plasma in the order 22:6 > 20:3> > 18:0 ~ 18:1 > 18:2 ~ 16:0. This is one of only a few reports using metabolomics to investigate single nutrient interactions in a human intervention study and highlights the potential of metabolomic technology in nutrition research in defining alternative functions for nutrients and elucidating how nutrients interact with metabolic pathways, in this case vitamin E.

## Abbreviations

BMI: Body mass index; EDTA: Ethylene diamine tetra acetic acid; HPLC: High pressure liquid chromatography; LC/MS: Liquid Chromatography Mass Spectroscopy; PCA: Principal component analysis; PLS-DA: Partial least squares-discriminant analysis; lysoPC: Lysophosphatidylcholine; Q^2^: Model predictive ability; QTOF: Quadrupole time of flight spectrometer; R^2^Y: Model goodness of fit; VIP: Variable influence on projection.

## Competing interests

The authors declare no competing interest.

## Authors’ contributions

JL conceived and designed the study and prepared the manuscript. MW carried out all the experimental work and statistical analysis and helped to draft the manuscript. All authors read and approved the final manuscript.
